# Symptomatic Sinus Bradycardia in a Patient With Acute Calculous Cholecystitis Due to the Cardio-Biliary Reflex (Cope’s Sign): A Case Report

**DOI:** 10.7759/cureus.25585

**Published:** 2022-06-01

**Authors:** Arjun Mainali, Samaj Adhikari, Tutul Chowdhury, Malavika Shankar, Nicole Gousy, Alix Dufresne

**Affiliations:** 1 Internal Medicine, Interfaith Medical Center, Brooklyn, USA; 2 Medicine, American University of Antigua, New York, USA; 3 Cardiology, Interfaith Medical Center, Brooklyn, USA

**Keywords:** cardiac autonomic activity, cardio-biliary reflex, copes sign, bradyarrythmia, acute calculus cholecystitis

## Abstract

Acute cholecystitis may present cardiovascular manifestation like cardiac ischemia leading to detailed cardiac workup without any obvious cardiac pathology. Here we describe a case who presented with typical signs and symptoms of cholecystitis exhibiting sinus bradycardia. This reflexive bradycardia was a result of autonomic vagal innervation and was resolved after cholecystectomy. This case highlights the importance of the cardio-biliary reflex and recommends clinicians ensure expedited management of cholecystitis to avoid unnecessary extensive cardiac workup.

## Introduction

The presentation of acute calculous cholecystitis can mimic signs and symptoms of cardiac ischemia. Furthermore, varying electrocardiographic findings have been reported in patients with calculous cholecystitis [1-3]. “Cope’s sign” is a well-established term in medicine that was initially reported by surgeon Zachary Cope in the medical literature [4,5]. Fewer cases of cardiobilliary reflex have been reported previously with the proposed mechanism being increased vagal tone [4-8]. This is the fourth case in literature in a patient diagnosed to have calculous cholecystitis found to have sinus bradycardia [4,8]. The clinical relevance in early diagnosis and appropriate management helps in minimizing unnecessary cardiac workup whenever the patient presents with acute abdominal pain with sinus bradycardia. We report a case of a patient initially presenting with acute abdominal pain and was also found to have severe sinus bradycardia. 

## Case presentation

A 37-year-old female with a past medical history of bronchial asthma, epilepsy, bipolar 1 disorder, and polysubstance abuse came to the emergency department with complaints of colicky right upper quadrant pain for one day associated with nausea and two episodes of nonbilious, non-projectile vomiting. She also reported a generalized feeling of weakness and lightheadedness for the same duration. She denied fever, diarrhea or constipation, yellowish discoloration of eyes, itching, anorexia, weight loss, or change in the stool and urine color. She also denied headache, confusion, syncope, chest pain, shortness of breath (SOB), palpitation, or dropped beats. She has a 14-year smoking history with a pack-year of seven. She is a social drinker and admitted to taking marijuana and clonazepam occasionally but she denied using any other illicit drugs. On review of her home medications, she was not on any heart rate (HR) lowering drugs. The family history was negative for cardiac disease, and all other histories were unremarkable.

**Figure 1:**
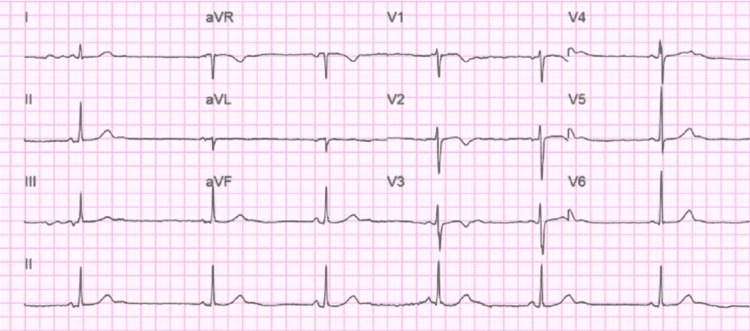
12 Lead ECG of the patient taken during admission ECG shows sinus bradycardia with an HR of 37/bpm HR, Heart rate; bpm, beats per minute

On physical examination, she was alert and in mild distress due to abdominal pain. Triage vitals showed HR of 35 beats/min, blood pressure: 151/72 mmHg, temperature: 36.5 C, and normal saturation on room air. Abdominal physical exam was significant for right upper quadrant tenderness and positive murphy’s sign but no signs of guarding and rigidity. On cardiovascular exam: S1/S2 normal, sinus bradycardia with normal rhythm with no murmur, rub, and gallop. The neurological exam was normal. The lab was significant for leukocytosis with a white blood cell (WBC) count of 12.9 K. All other lab parameters, including complete metabolic panel (CMP), thyroid-stimulating hormone (TSH), lipase, and high sensitivity troponin, were within normal limits. Urine toxicology was positive for cannabinoids and benzodiazepine. ECG showed HR of 37 beats/min, PR interval 138 ms, and QTc 416 ms with no significant ST/T wave changes (Figure 1).

**Figure 2:**
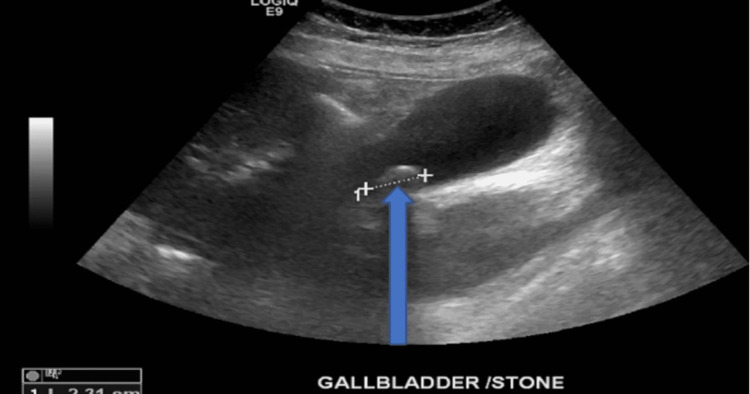
Abdominal ultrasound taken during patient admission Abdominal ultrasound is showing the gallbladder with a single solitary stone measuring 2.31 cm as indicated by the blue arrow cm, centimeter

She received atropine 0.5 mg in the emergency department (ED) with temporary improvement in HR up to 82 beats/min, however, it dropped down to 35-40 beats/min again in 1-2 hours. She was admitted to the cardiac care unit (CCU) for continuous cardiac monitoring and was evaluated for the cause of sinus bradycardia and abdominal pain. The echocardiogram was normal, with an ejection fraction (EF) of 60-65%. The lowest HR recorded during this episode was noted to be 32 beats/min on continuous cardiac monitoring; however, there were no features suggestive of hemodynamic instability such as a change in mentation, chest discomfort, shortness of breath, and hypotension, so atropine was not given during her CCU stay. Ultrasonogram (USG) abdomen showed cholelithiasis (Figure 2) but was inconclusive for acute cholecystitis.

**Figure 3:**
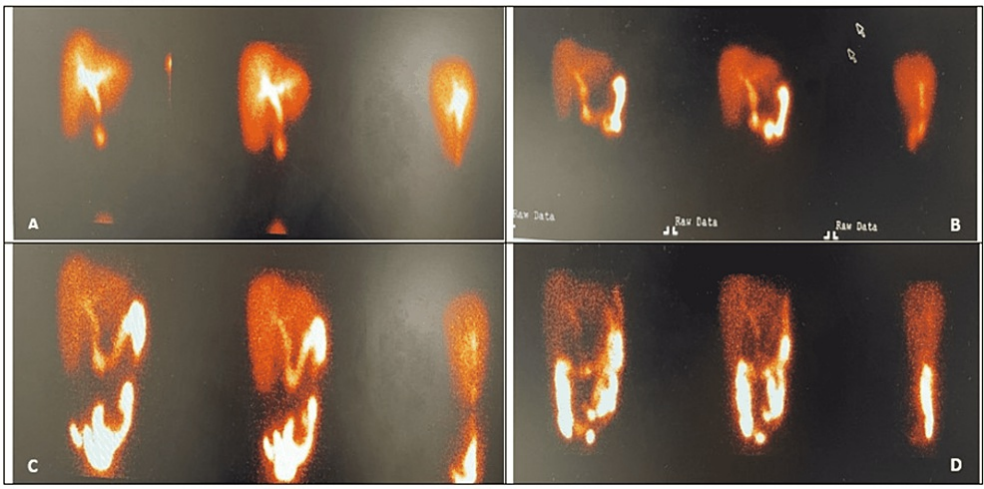
HIDA scan of the abdomen HIDA scan of the abdomen with static images at 30 minutes (A), 60 minutes (B), 2 hours (C), and 4 hours (D) after the administration of IV technetium 99m mebrofenin with nonvisualization of the gallbladder consistent with acute cholecystitis HIDA, hepatobiliary iminodiacetic acid; IV, intravenous

CT scan of the abdomen showed a distended gallbladder with cholelithiasis and was suggestive of early cholecystitis. She was started on the antibiotic piperacillin/tazobactam for cholecystitis and pain medication. Surgery was consulted and recommended for the hepatobiliary iminodiacetic acid (HIDA) scan for the definitive diagnosis. HIDA scan was positive for acute cholecystitis as described in (Figure 3).

**Figure 4:**
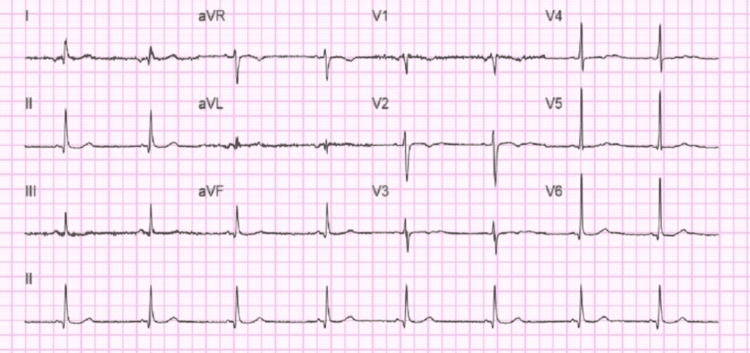
12 Lead ECG taken after cholecystectomy 12 Lead ECG taken after cholecystectomy showing sinus bradycardia with a heart rate of 49 beats per minute

She underwent Laparoscopic cholecystectomy on the third day of admission. Patient HR significantly improved after the surgery with postoperative ECG showing HR of 49, PR interval: 100, and QTc: 420 as in (Figure 4). 

Her HR continued to improve after the surgery, with HR recorded at 51-62 beats/min on continuous cardiac monitoring. On the fifth day of admission, she was discharged after she improved clinically with a resolution of her leukocytosis and was discharged with oral ciprofloxacin and metronidazole to complete seven days of antibiotics.

## Discussion

In 1971, O’Reilly and Krauthamer [5] described the development of reflex sinus bradycardia and other various electrocardiogram changes in the setting of acute cholecystitis or biliary colic, also known as Cope’s sign or the cardio-biliary reflex [4,9]. Although most pathologies relating to the gallbladder rely heavily on the clinical presentation for diagnosis, some patients may present with symptoms mimicking acute coronary syndrome (ACS) along with dynamic ECG changes suggestive of ACS [5,6]. Among these ECG changes common arrhythmias that can be seen include bradycardia, ST-segment elevation, T wave inversion, and AV nodal or right bundle branch block, with bradyarrhythmias being the most commonly seen [10]. The development of bradycardia is thought to be induced by the activation of two sets of autonomic vagus nerves in the T4 and T6 distribution, namely connecting the heart and gallbladder respectively [5,10,11]. This reflex is thought to be incited by increased pain and inflammation in the gallbladder, such as in acute cholecystitis, which leads to increased activation of autonomic neurons in the reflex arc inducing significant ECG changes [11]. 

Kaufman et al. [12] and other studies [3,9] have shown that when inflammation of the gallbladder occurs, vagal tone can be increased, and subsequently decreased, by the administration of intramuscular atropine, resulting in the normalization of any ECG changes. Additionally, patients with underlying cholecystitis did not have recurring ECG changes after the removal of their gallbladder [13]. This is consistent with the resolution of bradycardia seen with this patient after cholecystectomy. 

As with any patient with symptomatic or dynamically changing arrhythmias, special attention should be given to the patient’s cardiac enzymes. In the setting of gallbladder-related pathology resulting in the development of Cope’s sign, cardiac enzymes will remain within normal limits. This will help guide physicians in suggesting that the underlying cause for any acute ECG changes may be extracardiac in origin [3]. This study can help facilitate the diagnosis of underlying gallbladder disease and expedite treatment.

## Conclusions

The presentation of acute cholecystitis can prompt the physicians to further expedite the cardiac workup which delays the treatment of this life-threatening condition. Our patient responded well with reversal of sinus bradycardia after cholecystectomy which supports increased vagal tone causing sinus bradycardia initially. Clinicians need to be vigilant in the further management of patients presenting with sinus bradycardia who have concomitant acute abdominal pain.
